# Temporal Relationships Between Abdominal Pain, Psychological Distress and Coping in Patients With IBS – A Time Series Approach

**DOI:** 10.3389/fpsyt.2022.768134

**Published:** 2022-07-14

**Authors:** Felicitas Engel, Tatjana Stadnitski, Esther Stroe-Kunold, Sabrina Berens, Rainer Schäfert, Beate Wild

**Affiliations:** ^1^Department of General Internal Medicine and Psychosomatics, University of Heidelberg, Heidelberg, Germany; ^2^Department of Quantitative Methodology, University of Ulm, Ulm, Germany; ^3^Department of Psychosomatic Medicine, Division of Internal Medicine, University Hospital Basel, Basel, Switzerland

**Keywords:** irritable bowel syndrome, time series analysis, temporal relationships, psychological variables, somatic variables

## Abstract

**Objective:**

Irritable bowel syndrome (IBS) is a chronic disease leading to abdominal pain that is often related to psychological distress. The aim of the study was to investigate the temporal relationships between abdominal pain and psychological variables in patients with IBS.

**Methods:**

This longitudinal diary study included eight patients from a waiting group, recruited in the frame of a pilot intervention study. During their waiting time of 3 months the patients answered questions daily regarding somatic and psychological variables using an online diary. All patients were considered and analyzed as single cases. The temporal dynamics between the time series of psycho-somatic variables were analyzed using a vector autoregressive (VAR) modeling approach.

**Results:**

For all patients, positive same-day correlations between somatic and psychological time series were observed. The highest same-day correlations were found between somatic symptoms and pain-related discomfort (*r* = 0.40 to *r* = 0.94). Altogether, *n* = 26 significant lagged relationships were identified; *n* = 17 (65%) indicated that somatic values were predictive of psychological complaints on the following days. *N* = 9 (35%) lagged relationships indicated an opposite relationship in that psychological complaints were predictive of somatic symptoms. Three patients showed a significant positive same-day correlation between abdominal pain and use of a positive coping strategy. However, significant lagged relationships in two patients showed that for these patients the use of positive thinking as a coping strategy was unhelpful in reducing pain on the following days.

**Conclusions:**

In patients with IBS abdominal symptoms appear to be closely related to psychological symptoms. For some patients, somatic complaints predict psychological complaints, in other patients the directionality is opposite. The impact of coping strategies on somatic symptoms varies among patients, therefore their role for a possible reduction of pain should be further explored. The results suggest the need of characterizing patientsindividually for effective health interventions. Individual time series analyses provide helpful tools for finding reasonable person-level moderators.

## Introduction

Irritable bowel syndrome (IBS) affects about 8% of the European population (1) and is most recently understood as a disorder of (microbiota-) gut-brain interaction (2, 3) with a multifactorial origin that includes biological, (epi-)genetic, psychological, and social factors (4, 5). Patients with IBS suffer from recurrent abdominal pain that is associated with a change in frequency or form (appearance) of stool and can be related to defecation (6). Currently, the symptom pattern is not sufficiently explained by peripheral organ pathology. Many patients who suffer from IBS also suffer from comorbid, depressive, or anxiety-related disorders (5, 7–9).

A possible upper-level mechanism linking IBS with comorbid disorders could be a genetic alteration of serotonin reuptake receptors in some patients with IBS (10–12). Some of these alterations can affect bowel motility and have been shown to be associated with depression and anxiety (10). On the physiological level, the underlying mechanism for the close association of somatic and psychological symptoms could be explained by the concept of the (microbiome-) gut-brain axis. The (microbiome-) gut-brain axis is a complex network of connections between the microbiota, the enteric nervous system, and the central nervous system (2, 13). Psychological stress can lead to an activation of the autonomic system, the HPA-Axis, or the immune system resulting in increased IBS symptoms (5, 14). For example, psychological stress has been shown to lead to altered motility, secretion, and barrier function *via* mast cell activation (14). Furthermore, psychological stress can lead to catastrophizing, illness worries and increased (negative) attention to normal gut signals which are then perceived as threatening and thus reinforced in a vicious circle (5).

Results from population-based studies indicate a bidirectional interaction between IBS and both depressive and anxiety-related symptoms (9, 15). However, it still remains unclear how the psychological complaints are temporally related to somatic symptoms. A recent study using an experiential sampling method (ESM) in IBS patients showed that bowel symptoms temporally predicted negative affect but counter-intuitively daily life stress predicted a decrease in abdominal pain (16). However, this study covered a time period of only 14 days. Another study found that stress and gastrointestinal symptoms were strongly associated in the same week, but no direct relationship between stress in the first week and gastrointestinal symptoms in the following week (17). Furthermore, both studies focused on the mean values from a larger patient sample; the various relationships in individual patients may therefore not have been reflected in the aggregated data analysis.

Another interesting topic in IBS patients is the interaction between IBS symptoms and coping strategies. A previous study showed that the use of coping strategies would be connected with gastrointestinal and extraintestinal symptom severity (18). In addition, in IBS patients' levels of coping resources and catastrophizing appear to be associated with gastrointestinal symptom severity (18, 19). It is also reported that IBS patients would use passive coping strategies more frequently (such as escape-avoidance strategies instead of intended problem solving) compared to healthy controls (20). Here too, it would be interesting to determine to what extent coping strategies are temporally related to IBS complaints and whether or not they are able to influence IBS complaints. In summary, IBS symptoms and psychological distress show a mutual relationship; coping strategies are assumed to influence the up- and down-regulation of IBS symptoms. However, longitudinal data is necessary in order to model the temporal interactions between IBS symptoms and psychological complaints on an individual level using time series modeling. First results from an own previous single-case study indicated a high correlation between somatic and psychological complaints with somatic symptoms temporally preceding psychological complaints. Also, a patient's positive thinking as a coping strategy appeared to be helpful in reducing the pain (21).

To date, there are various theoretical approaches to symptom development and worsening in IBS in general. However, patients with IBS show a large variability regarding biopsychosocial variables. An idiographic approach, based on multiple repeated measurements within a single case, has the advantage to detect individual patterns and enable patient-tailored advice.

The aim of the present study was to analyze the temporal relationships and interactions between somatic and psychological complaints of eight patients with IBS in the frame of a time series design. In addition, for these particular patients, the impact of personal coping strategies on abdominal pain was explored.

## Materials and Methods

### Study Design

The present study included eight patients (diagnosed with IBS by clinicians and ROME-III criteria), assessed by using a longitudinal, observational design. ROME-III criteria were collected by a questionnaire and validated in a telephone interview prior to patient inclusion. According to ROME-III an IBS can be diagnosed when a patient has recurrent abdominal pain or discomfort at least 3 days per month associated with 2 or more of the following: (1) improvement with defecation, (2) onset associated with change in frequency of stool, (3) onset associated with a change in form (appearance) of stool. The criteria must be fulfilled for the last 3 months with symptom onset at least 6 months prior to diagnosis (27). Diagnoses regarding food malabsorption and intolerance were made by the treating physicians and based on breath/clinical tests with the corresponding clinical history. In the case of malabsorption/intolerance, patients had been advised to follow an appropriate diet. Since the patients did not show remission from symptoms under this diet the diagnosis of IBS was made or maintained.

All patients were recruited by way of an appointment in the outpatient specialty clinic of the Department of General Internal Medicine and Psychosomatics, University Hospital Heidelberg, for functional gastrointestinal disorders, before the start of a pilot intervention (28, 29).

Written informed consent was obtained; the study was approved by the medical ethics committee of the University Hospital Heidelberg.

In the course of their waiting time patients answered questions daily using an online diary that included somatic, psychological, and coping variables. All patients filled in the online diary over a period of 3 month between 07/2014 and 05/2015 (different start times). The number of measurement days per patient varied between 68 and 94, thus fulfilling the requirements for the application of a comprehensive time series analysis approach (30).

### Online Diary Measurements

Patients were given information about the online diary and were asked to complete it daily (between 4 and 12 p.m.). The diary questions referred to questions from validated questionnaires, shortened so that a completion time of 5–10 min could be achieved. A visual analog scale (VAS) with bipolar levels was used by the patients and later transcribed into a numeric scale (1–101). In addition, patients were provided an opportunity to write a short free text in the diary.

The items included in the analysis are shown in Table 1 (21).

**Table 1:** List of Online-Diary Items included in the time series analysis.

**Somatic variables**	**Items implemented in the online diary**
Abdominal pain (AP)	“How severe is your abdominal (tummy) pain”
	→ Adapted from the irritable bowel severity scoring system (IBS-SSS) (22)
IBS associated daily impairment (DI)	“Please indicate how much your irritable bowel syndrome is affecting or interfering with your life today”
	→ Adapted from the irritable bowel severity scoring system (IBS-SSS) (22)
**Psychological variables**	**Items implemented in the online diary**
Nervousness (N)	“Today, how much were you distressed by nervousness or shakiness inside?”
	→ Adapted from the brief symptom inventory (BSI) (23)
Tension (T)	“Today, how much were you distressed by feeling tense or keyed up”
	→ Adapted from the brief symptom inventory (BSI) (23)
Depressiveness (D)	“Today, how often have you been bothered by feeling down, depressed, or hopeless?”
	→ Adapted from the Patient-Health-Questionnaire (PHQ) (24)
Pain associated discomfort (PD)	“Today, how much have you been bothered by stomach pain”
	→ Adapted from the Patient-Health-Questionnaire (PHQ) (25)
**Coping strategies**	**Items implemented in the online diary**
Catastrophizing (C)	“Today, when experiencing IBS-pain you had the feeling that you couldn't go on“
	→ Adapted from the coping strategies questionnaire (CSQ) (26)
Hopelessness (H)	“When you had IBS-pain today, you thought: “It's terrible and I feel it's never going to get any better”
	→ Adapted from the coping strategies questionnaire (CSQ) (26)
Coping: positive thoughts (CPT)	“Today, when experiencing IBS-pain I thought of things I enjoy doing”
	→ Adapted from the coping strategies questionnaire (CSQ) (26)
Coping: Imagining pain outside the body (CIP)	“When experiencing IBS-pain, today I imagined that the pain is outside of my body”
	→ Adapted from the coping strategies questionnaire (CSQ) (26)

*This table was also included in Engel et al. (21)*.

### Patient Descriptions

Table 2 shows demographic and clinical characteristics of the included patients.

**Table 2:** Demographic characteristics of the study sample.

	**IBS patients (*n* = 8)**
IBS diagnoses (*n*;%)	
Mixed-type (IBS-M)	4 (50.0)
Diarrhea-dominant (IBS-D)	3 (37.5)
Unsubtyped (IBS-U)	1 (12.5)
Age (MW ± Std)	35.37 ± 11.86
Sex (*n*; %)	
Male	3 (37.5)
Female	5 (62.5)
Marital Status (*n*; %)	
Single	5 (62.5)
Married	3 (37.5)
Living situation (*n*;%)	
living alone	2 (25.0)
living in a partnership	5 (62.5)
living with parents	1 (12.5)
Education (*n*; %)	
≤ 8 years	0 (0.0)
9–11 years	2 (25.0)
>11 years	6 (75.0)

In the following, we briefly describe the patients including their disorder history, comorbidities, and the free-text diary data in order to provide some information for the interpretation of the individual time series analyses. All patients were German and can be described as typical patients with IBS treated in our outpatient clinic: They suffer from various somatic and psychological comorbidities and show a high burden of complaints (28).

**P1** was a man in his late 40s, employee, suffering from diarrhea-dominant IBS and gluten-sensitivity (no celiac disease) for 2 years. His symptoms had begun following a career change that was associated with increased stress levels. He reported that he was under a high pressure to perform - also reflected in the diary data. Several peaks of abdominal pain (e.g., day 9/10, day 12–16, day 21/22) were related to an actual high stress situation on his job. In addition, episodes of less or no pain were associated with periods when the patient was on vacation and /or thinking about other things than work (days 54–65). Other stressors (such as a sick child or trying gluten) appeared not to be associated with pain.

**P2** was a woman in her late 20s, employee, suffering from diarrhea-dominant IBS (main symptom pain), fructose malabsorption, depression, and social phobia. Fructose malabsorption is common in IBS and does not rule out the diagnosis of irritable bowel syndrome, particularly when improvement is not obtained following an appropriate diet (31–33). Of note is that this patient P2 had a generally high abdominal pain and daily impairment in her self-report data with almost no fluctuation. She described her life as very stressful: an exhausting work and numerous social obligations. Although the patient canceled many activities, she necessarily increased her activities in times of life events such as the death of a close relative (day 46). Apart from the IBS-pain, she described many additional, almost daily somatic problems such as severe sleeping disturbances and headaches, as well as pain in different areas of her body. She visited medical doctors on a frequent basis. In the course of these consultations, she received various diagnostic procedures, but no psychological treatment.

**P3** was a woman in her early twenties, employee, suffering from mixed-type IBS for 7 years, lactose intolerance, and sorbitol intolerance. She had been diagnosed with a panic disorder in the past. In the free diary text she described, almost exclusively, positive feelings and positive events. Even during times with higher abdominal pain (e.g., days 17–34) she described how good everything was—apart from the symptoms. However, in her contact to our outpatient clinic she also assumed a connection between IBS-symptoms, stress, tension and regrets regarding her work.

**P4** was a man in his late 30s, employee, with mixed-type IBS, sorbitol intolerance, suspected wheat/gluten-sensitivity, reflux esophagitis, and a depression diagnosis. The patient used the free text for only the first 5 days, describing his work as challenging. During the appointment in our outpatient department he described an inner restlessness and nervousness. He generally attributed improvements in health to natural remedies and nutritional advice.

**P5** was a woman in her early 20s, student, with mixed-type IBS, functional dyspepsia, lactose intolerance, bronchial asthma, depression, and suspected emotionally unstable personality disorder. An atypical eating disorder had also been discussed. She suffered from IBS for several years and described ongoing fears of being unable to cope with her symptoms. She was very afraid of bodily changes (e.g., a bigger belly due to flatulence or gaining weight due to being unable to participate in an intensive sport program because of her symptoms) and of other people's reactions. In the free text of the diary she expressed her impairment and insecurity also reflected in the data by high pain scores or high daily impairment (e.g., days 1, 17, 33, episode about days 40–50, 60).

**P6** was a woman about age 30, employee, with mixed-type IBS, fructose malabsorption, and tension headache. Since attending school, she had symptoms that had worsened over the past years. She described feelings of social anxiety in relation to her complaints that were also reflected in the free text of her diary. Her fear and shame were related to other people, who, she feared, might notice that something was wrong (e.g., because of stomach noises). Apart from this anxiety, she described an increase in symptoms due to menstrual cramps (day 15), and problems at work (day 16). Interestingly, her time series indicated an improvement regarding IBS symptoms and daily impairment when she described almost no positive events (except for day 11, – at the beginning of the weekend).

**P7** was a woman about age 50, employee, with unsubtyped IBS (IBS-U, main symptom: bloating), sorbit malabsorption, fibromyalgia, and depression. Symptoms had started a long time ago with an onset of more severe symptoms approximately 7 years ago. She described that her complaints were aggravated by nervousness and gas-forming food. Additional stress factors, as described, were a difficult childhood and an unfulfilled desire to have children. In the free text of the diary she frequently reported complaints such as tiredness, pain (abdominal, leg, menstrual pain), and bloating as well as having negative feelings (e.g., everything was too exhausting) She very rarely reported positive feelings.

**P8** was a man about age 50, unemployed, on sick leave for several months, with diarrhea-dominant IBS, lactose intolerance, and severe depression. He described that symptoms began more than 20 years ago and that they could be controlled only minimally by medication (loperamide). He completed the free text diary for only the first few days. He indicated better episodes such as symptom improvement of diarrhea, anxiety, or depression (days 1, 3, 8) and worse episodes due to ineffective medication (bifidobacterial, day 6) or thinking about problems (day 14).

### Statistical Analysis

The analyses were conducted using the R software. For further descriptions, more elaborate explanations regarding time series analysis, and implementations of all analyses with the R software we refer to further literature (30, 34, 35). Initially, the following analyses were conducted for each time series: graphical examinations; calculations of descriptive statistics (range, median, mean, standard deviation) and autocorrelation functions (ACF); tests for stationarity with the augmented Dickey–Fuller (ADF) procedure. Subsequently, cross-correlation functions (CCF), instantaneous correlations, and simultaneous regressions with psychological measures as dependent variables and somatic variables as predictors were estimated. Temporal interdependencies between time series were analyzed using a vector autoregressive (VAR) modeling approach. The VAR methodology investigates the temporal dynamics between two or more time series by separating the time-lagged relations from the simultaneous ones. The VAR technique, therefore, allows inferences regarding the temporal order of the effects employing the causality concept introduced by Granger. The essential idea of the Granger causality test is that if current and lagged values of a time series X improve prediction of future values of a series Y, the former series Granger-causes the latter. Furthermore, the VAR approach can handle time series that mutually influence each other, thereby revealing feedback effects. The behavior of a VAR system is described using impulse response (IRA) analyses and forecast error variance decompositions (FEVD). Impulse response functions (IRF) examine interdependencies within a VAR system by tracing the effect of an exogenous shock in one of the series on other variables. The FEVD estimates the amount of variance in each variable that can be explained by other variables of the system during a specific period.

## Results

**Figure 1:**
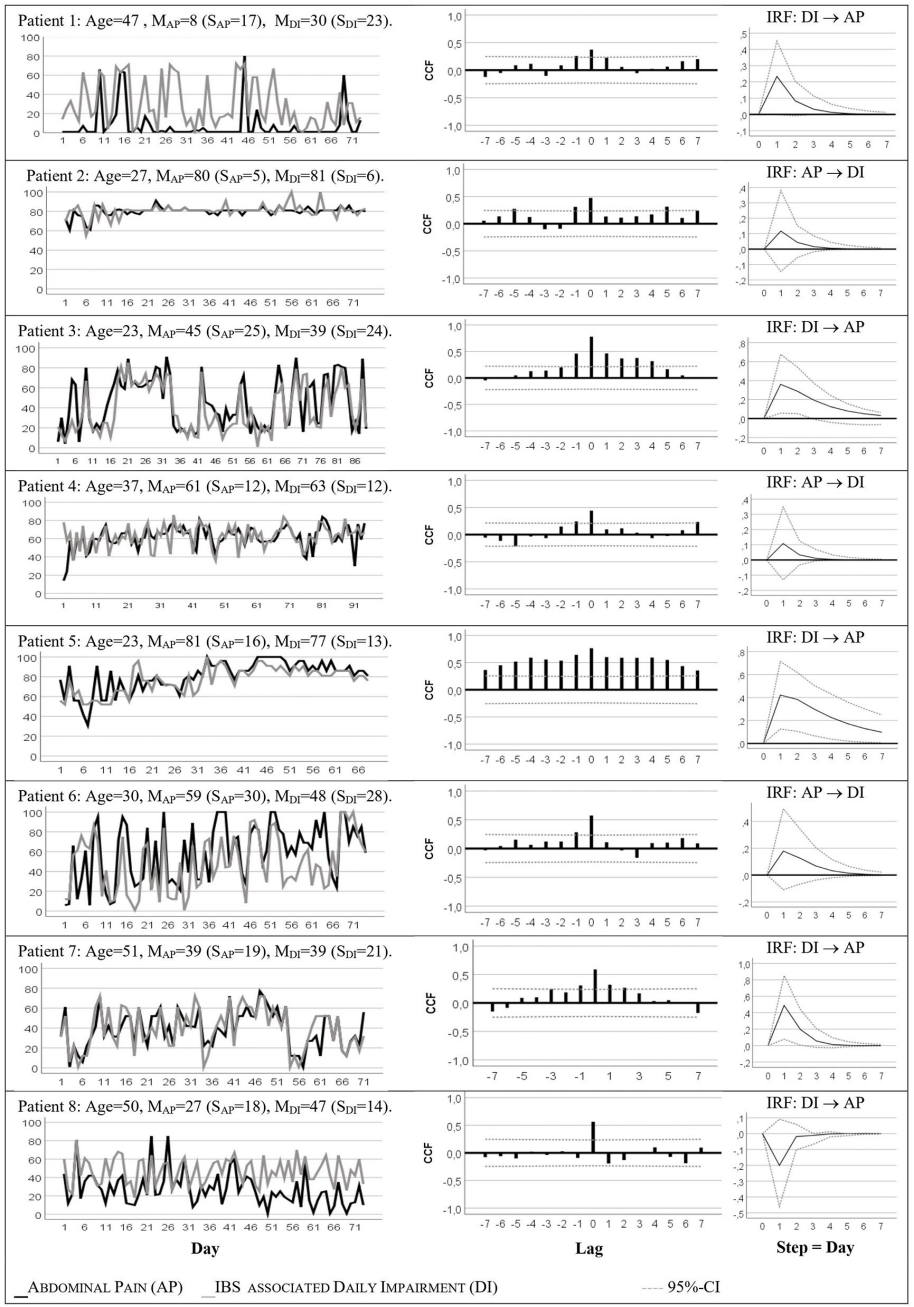
Somatic Time Series of Abdominal Pain (AP) and IBS Associated Daily Impairment (DI) with their Cross-Correlation (CCF) and Impulse Response Functions (IRF).

The left part of Figure 1 visualizes developments of somatic symptoms, abdominal pain (AP), and IBS associated daily impairment (DI) of the patients; observation periods varied from 68 to 94 successive days. The patients showed various patterns of discomfort. The mean values ranged from mean (AP) = 8 to mean (AP) = 81 and mean (DI) = 30 to mean (DI) = 81. The time series of patients 1, 3, 6–8 showed high variability (standard deviation, s = 17 to s = 30) due to the alternation of strong and slight perceived discomfort. In contrast, AP and DI reports of Patient 2 were constantly high: mean (AD) = 80 [s (AD) = 8]; mean (DI) = 81 [s (DI) = 6]. All series exhibited no trends and were therefore stationary, with the exception of patient 5 where mean values of the first 30 days were distinctly lower than the average measures of subsequent observations.

The right section of Figure 1 shows the cross-correlation functions (CCF) and impulse response functions (IRF) of the time series AP and IBS associated daily impairment (DI). For all patients, the time series of AP and DI showed significantly positive same-day correlations that varied between *r* = 0.40 and *r* = 0.80. This means that for all patients, high values in abdominal pain were associated with high values in DI on the same day.

For four patients (P1, P3, P5, P7) the IRFs indicated significant temporal relations between somatic time series. For example, abdominal pain measures of patient 7 depended linearly and positively on previous day values of IBS associated daily impairment: Step 1 IRF=0.49, 95% CI=(0.08; 0.85).

The free text of the patients' diaries is mainly reflected in the time series data (for further information also see the case reports).

### Interdependencies Between Somatic and Psychological Time Series

Table 3 shows same-day correlations between the somatic and psychological time series (including negative coping). For all patients, somatic symptoms were most strongly related to pain-associated discomfort: *r* = 0.40 to *r* = 0.97. The amount of explained variance (R^2^) from linear regressions, with psychological measures as dependent variables and somatic variables as predictors, varied from 2 (patient 2) to 94% (patient 7, explained variance in pain associated discomfort).

**Table 3:** Significant instantaneous correlations between somatic (AP, DI) and psychological variables with the portion of explained variance in psychological variables (*R*^2^).

		**N**	**T**	**C**	**H**	**D**	**PD**
Patient 1
*(*N = 73)	AP	0.26	–	0.21	0.33	–	0.63
	DI	0.48	0.46	0.21	0.34	0.36	0.40
	*R^2^*	*0.24*	*0.22*	*0.07*	*0.17*	*0.13*	*0.43*
Patient 2
*(*N = 74)	AP	–	–	0.56	0.27	0.32	0.70
	DI	–	0.36	0.45	–	0.40	0.67
	*R^2^*	*0.02*	*0.13*	*0.36*	*0.08*	*0.18*	*0.65*
Patient 3 *N* = 89	AP	–	0.24	0.54	0.37	0.22	0.93
	DI	0.25	0.22	0.49	0.39	–	0.80
	*R^2^*	*0.06*	*0.06*	*0.30*	*0.16*	*0.05*	*0.87*
Patient 4 *N* = 94	AP	0.24	0.46	0.32	–	0.26	0.73
	DI	0.39	0.34	0.31	0.26	0.39	0.48
	*R^2^*	*0.16*	*0.24*	*0.14*	*0.07*	*0.16*	*0.56*
Patient 5 *N* = 68	AP	0.25	–	0.54	0.55	–	0.95
	DI	0.46	–	0.55	0.52	0.34	0.81
	*R^2^*	*0.24*	*0.04*	*0.33*	*0.33*	*0.17*	*0.91*
Patient 6 *N* = 74	AP	–	0.23	–	0.37	–	0.79
	DI	0.28	–	0.36	0.45	–	0.50
	*R^2^*	*0.08*	*0.06*	*0.13*	*0.22*	*0.02*	*0.62*
Patient 7 *N* = 71	AP	0.21	–	0.59	0.39	0.22	0.97
	DI	0.34	0.22	0.67	0.49	0.27	0.86
	*R^2^*	*0.14*	*0.06*	*0.45*	*0.24*	*0.07*	*0.94*
Patient 8 *N* = 73	AP	–	–	0.41	0.34	–	0.76
	DI	0.34	–	–	–	0.25	0.40
	*R^2^*	*0.15*	*0.09*	*0.26*	*0.12*	*0.09*	*0.58*

*R^2^is the portion of explained variance in psychological variables*.

*R^2^ from the regressions PV_t_ = β_1_SV1_t_+β_2_SV2_t_+e_t_ where PV, psychological variable; SV = somatic variable; AP = Abdominal Pain; DI, IBS Associated Daily Impairment; N, Nervousness; T, Tension; C, Catastrophizing; H, Hopelessness; D, Depressiveness; PD, Pain associated discomfort*.

### Lagged Relationships Between Somatic and Psychological Time Series

Table 4 summarizes the significant results of the VAR analyses for interdependencies between somatic symptoms and psychological complaints (including negative coping strategies). Twenty-six significant lagged or temporal relations were identified; 17 (65%) showed the direction “Somatic Symptoms → Psychological Variable” with 4 to 16 % of explained variance; nine (35%) demonstrated the direction “Psychological Variable → Somatic Symptoms” with 6 to 18 % of explained variance. For instance, for Patient 1, higher abdominal pain was followed by a delayed increase in depression (+0.30 standard deviations) on the next day; conversely, more hopelessness and pain-associated discomfort resulted in higher perceived abdominal pain the next day (+0.29, +0.51 standard deviations). Interestingly, for P7 and P8 the lagged influence of somatic symptoms on psychological variables showed a different quality compared to the other patients. For P7, an increase in abdominal pain on one day was followed by a decrease in nervousness and tension. Similarly, for P8, an increase in DI was followed by decreased nervousness and depression at 2 or 3 days later.

**Table 4:** Lagged dependencies between somatic and psychological variables.

**Patient**	**Type of dependency**	**VAR**	**Strongest Impulse-Response**	**Granger causality Test**		**% FEVD**
		**Order**				***h*** **= 10**
				**F**	**p**	
1	Abdominal Pain → Depressiveness	1	+0.30	6.63	0.011	9
	Hopelessness → Abdominal Pain	1	+0.29	5.99	0.016	8
	Pain associated discomfort → Abdominal Pain	1	+0.51	13.2	<0.01	17
2	Abdominal Pain → Depressiveness	2	+0.23	4.33	0.015	7
	Catastrophizing → Daily Impairment	1	+0.40	11.1	0.001	14
3	Abdominal Pain → Catastrophizing	4	+0.36	3.20	0.015	16
	Catastrophizing → Abdominal Pain	4	−0.41	2.91	0.024	18
	Daily Impairment → Pain associated discomfort	1	+0.33	4.35	0.038	6
4	Abdominal Pain → Catastrophizing	1	+0.26	6.33	0.013	7
	Abdominal Pain → Depressiveness	1	+0.22	4.36	0.038	4
	Tension → Abdominal Pain	3	+0.16	2.73	0.046	9
5	Abdominal Pain → Nervousness	3	+0.30	2.72	0.047	9
	Daily Impairment → Nervousness	1	+0.32	5.92	0.016	9
	Daily Impairment → Hopelessness	1	+0.30	5.14	0.025	9
	Daily Impairment → Pain associated discomfort	1	+0.41	7.68	<0.01	13
	Pain associated discomfort → Abdominal Pain	1	+0.62	4.45	0.037	6
6	Daily Impairment → Hopelessness	1	+0.34	7.39	<0.01	9
	Nervousness → Daily Impairment	1	−0.23	4.18	0.043	6
7	Abdominal Pain → Nervousness	2	−0.33	4.18	0.018	10
	Abdominal Pain → Tension	2	+0.18	4.40	0.014	9
	Depressiveness → Abdominal Pain	1	+0.26	5.16	0.025	9
	Daily Impairment → Tension	2	−0.20	4.96	0.008	9
	Daily Impairment → Pain associated discomfort	1	+0.52	5.42	0.021	8
8	Daily Impairment → Nervousness	2	−0.36	6.91	0.001	14
	Daily Impairment → Depressiveness	3	−0.32	4.56	0.005	13
	Depressiveness → Daily Impairment	3	+0.47	2.69	0.049	13

*A significant Granger test implies that the first variable has causal impact on the second variable. The test statistics is F (df_1_,df_2_), where df_1_ is a number of tested restrictions (k) and df_2_ = 2T−4k−2 for bivariate VAR models, T is length of time series, k is order of VAR model. Forecast Error Variance (FEV) Decomposition estimates the amount of variance in a dependent variable that can be explained by a corresponding cause variable during a period h*.

### Lagged Relationships Between Somatic Variables and Positive Coping Strategies

Table 5 shows the dependencies between somatic variables and positive psychological coping strategies.

**Table 5:** Dependencies between somatic variables and psychological coping strategies.

**Patient**	**Type of dependency** **( → lagged, ↔instantaneous)**	**Significant** **Instantaneous** **Correlation**	**VAR** **Order**	**Strongest** **Impulse-** **Response**	**Granger** **causality** **Test**		**%** **FEVD** ***h* = 10**
					**F**	**p**	
1	Abdominal Pain → CPT	0.42	1	+0.30	6.70	0.011	8
2	Daily Impairment → CIP	–	1	+0.16	11.9	<0.01	17
3	Abdominal Pain → CPT	0.33	1	+0.25	5.35	0.022	7
	Daily Impairment → CPT	0.41	1	+0.32	7.88	<0.01	12
	Daily Impairment → CIP	0.26	2	+0.30	4.12	0.018	6
4	Abdominal Pain ↔CPT	–	–	–	–	–	–
	Abdominal Pain → CIP	–	1	+0.25	5.95	0.016	5
5	Abdominal Pain → CPT	–	1	−0.28	4.89	0.029	8
	Daily Impairment → CPT	–	1	−0.34	7.37	<0.01	11
	CPT → Abdominal Pain	–	1	+0.22	5.23	0.024	5
6	Abdominal Pain → CPT	–	1	−0.19	3.37	0.068	4
7	CPT → Abdominal Pain	–	1	+0.24	4.29	0.042	6
8	Abdominal Pain ↔CPT	0.25	–	–	–	–	–

*CPT =“Coping: Positive thoughts” and CIP = “Coping: Imaging pain outside the body.”*

*A significant Granger test implies that the first variable has causal impact on the second variable. The test statistics is F (df_1_,df_2_), where df_1_ is a number of tested restrictions (k) and df_2_ = 2T−4k−2 for bivariate VAR models, T is length of time series, k is order of VAR model. Forecast Error Variance (FEV) Decomposition estimates the amount of variance in a dependent variable that can be explained by a corresponding cause variable during a period h*.

For three of the patients (P1, P3, P8), significant positive same-day correlations were observed: *r* = 0.25 to *r* = 0.42. For four patients (P1–P4), somatic symptoms were followed by more coping next day with 5 to 17% of explained variance. In only two patients (P5, P7,) the use of coping strategies was temporally predictive for abdominal pain on the next day. However, and unexpectedly, for these patients a more frequent use of coping strategies was followed by an increase in abdominal pain on the next day (with 5–6% of explained variance in AP).

## Discussion

This study investigated the daily temporal relationships between somatic and psychological variables in patients with IBS. Of note is the fact that in some aspects the patients presented quite similar patterns in cross-correlations whereas in other aspects the results differed widely across patients. Therefore, the patterns that were noted for the majority of patients will be reviewed first, while the individual patterns of illness and coping will be discussed afterwards.

The somatic time series of all patients (except P5) remained stationary during the observation period - that is, time series exhibited no trends in this study period where the patients were waiting for the beginning of a group program and did not receive additional psychotherapeutic treatment. This corresponds to previous studies describing IBS as a chronic disease. Only 55% of IBS-patients report a remission over a period of more than 10 years (36).

For all patients, strong positive same-day correlations of abdominal pain (AP) and IBS-associated daily impairment (DI) were observed. Not only does this result reflect the strong association between pain and daily impairment, but it also indicates that the self-reported diary scores are valid. For P1, P3, P5, and P7 the temporal relationship between AP and DI were significant in the direction that a higher symptom score in DI was followed by a higher AP score; thus, in 50% of the patients, higher daily impairment appears to trigger higher abdominal pain. For P2, P4, and P6, the direction of the temporal relationship between AP and DI was the reverse, but only as a trend.

All patients showed high positive same-day correlations between IBS associated daily impairment and psychological variables such as nervousness, depressiveness, or hopelessness. We can therefore conclude that all of the included IBS patients were psychologically burdened on days with higher somatic symptoms. In addition, for all patients, somatic symptoms were most strongly related to pain-associated discomfort. This may confirm the validity of the diary entries.

Regarding the temporal relationships between somatic symptoms and coping strategies, we were unable to replicate the results of a previous single case analysis for all patients (21). Only two patients (P5, P7) showed a significant temporal dependency between the use of a positive coping strategy on one day and abdominal pain on the following day. However, in our previous single case study the use of a specific coping strategy was followed by a decrease in pain on the subsequent day - whereas for the two patients in this present study, the association was opposite: the intensified use of a positive coping strategy was followed by an increase in pain on the following day. We therefore cannot conclude that the use of coping strategies is, generally speaking, beneficial for IBS patients.

Further, for all patients, significant temporal relationships were found between somatic and psychological variables. In 6 patients, an increase in psychological variables such as nervousness or tension significantly predicted an increase in abdominal pain or irritable bowel related somatic impairment on the following days. For two patients the opposite was true: here, higher scores in psychological variables predicted a decrease in pain or somatic impairment on the following days. These results are partly in contrast to a previous study using ESM in IBS patients reporting that daily life stress predicted a decrease in abdominal pain (16). Vice versa, in our study, for 6 patients, increased abdominal pain or IBS related impairment on one day predicted an increase in psychological variables (such as depressiveness or tension) whereas for one patient the effect was reversed and for one patient, a mutual relationship was found. These results correspond largely to the results of the ESM study that showed a significant temporal relationship between increased IBS symptoms on one day and increase in negative affect on the following day. However, the ESM study measured stress and IBS symptoms over a time period of only 14 days and analyzed moment-to-moment associations between the variables and, therefore, results are not easily comparable. Another study that measured stress and bowel symptoms over a period of 4 weeks found no significant independent temporal relationship between stress and IBS symptoms (17). However, this study used structural modeling for data analysis and thus determined pathways back and forth over the weeks that are more complex to understand than the temporal relationship between two variables.

In our study, the direction of the temporal relationship between somatic and psychological variables varied across patients and individual patterns were clearly visible.

In his diary free text P1 expressed the insight that the severity of his IBS symptoms could be related to stress. This was reflected in the time series data by high same-day correlations between DI and tension as well as DI and nervousness. For this patient, higher AP on 1 day was followed by higher depressiveness on the next day. However, there appeared to be a mutual relationship between somatic and psychological variables because a higher score in hopelessness was followed by higher IBS related impairment. The time series data of AP and DI showed clear peaks of increased discomfort that decreased shortly afterwards.

Results for P2 showed a high same-day correlation between somatic complaints and catastrophizing. In her case, abdominal pain was followed by depressiveness, while higher catastrophizing predicted higher daily impairment on the following days. Interestingly, this patient reported visiting her general practitioner, as well as various medical specialists quite frequently. According to her story, the doctors continued to give her diagnostic information instead of introducing another perspective of IBS symptoms. This approach is often used by doctors who frequently experience patients with medically unexplained physical symptoms as difficult (37). However, too many diagnostic procedures with a focus on somatic complaints will probably result in an iatrogenic somatic fixation in these patients (38). Studies show that cognitive calming instead of further diagnostics can have a positive effect on the patient and thereby reduce the use of health care (39). In addition, psychosocial support and psychotherapy appears to be helpful (40).

P3 described, almost exclusively, positive feelings and events. However, we found a mutual relationship between catastrophizing and pain. A higher pain score predicted more catastrophizing at 4 days later whereas a higher catastrophizing score was followed by less pain at 4 days later. For this patient, one could hypothesize that admitting negative feelings could lead to more self-acceptance and relief. Exaggerated positive thinking can be a denial, thus avoiding measures to reduce pain and relieve stress. Furthermore, the suppression of emotional expression and unwanted thoughts have been shown to be correlated to higher physiological arousal and psychopathology (41).

P4 was a patient who mainly attributed his complaints to physical causes. However, his diary data showed a strong same-day correlation between tension and somatic symptoms. In addition, increased tension was followed by stronger pain at 3 days later. This could reflect an inability to link psychological processes to physical ones and vice versa; this is often observed in IBS (42).

P5 was a patient who was completely focused on her body. Her symptoms and her fears (e.g., of getting a bigger belly) could also be signs of an eating disorder. Comorbid eating disorders are common in patients with functional gastrointestinal diseases (up to 10% in our outpatient clinic) (28); a connection between these disorders and higher levels of depression is possible (43, 44).

P6 reported profound social fears in relation to her IBS symptoms. Her diary data showed high same-day correlations between somatic symptoms and hopelessness in addition to catastrophizing. Interestingly, in this patient an increase in nervousness was followed by a decrease in DI on the next day. In the free text the patient frequently described social anxiety and the specific fear that, in social contexts, she would have to rush to a toilet. The latter is often described by IBS patients (45) and may lead to social avoidance, which, in turn, may increase social anxiety. Furthermore, many patients with IBS are known to be afraid of eating or avoid eating in social situations because eating may induce or exacerbate their symptoms (46, 47). This can lead to weight loss, eating disorders, or further psychosocial impairment (48). By addressing fears and avoidance, psychotherapy could be helpful for these patients.

P7 was aware of the connection between her life stressors and (the following) somatic complaints. This was reflected in the time series data by high same day correlations between somatic complaints and psychological variables. This patient also had a diagnosis of depression - also common among IBS patients. Her diary data showed that higher depressiveness on one day was followed by an increase in pain on the next day; conversely, a higher score in pain on one day was followed by a decrease in nervousness 2 days later. One could thus speculate that the pain symptoms played a part in the mood regulation of the patient.

P8 was on sick leave for several months; the comorbid diagnosis of depression contributed to his symptom burden. Interestingly, for this patient, time series analyses showed that increased daily impairment was followed by decreased depressiveness and nervousness at 4 days later. A previous study found that more than 40% of the patients on longer sick leave reported feelings of guilt (49). It is possible that for this patient, the experience of stronger somatic symptoms was connected with a justification for not being able to work – thereby resulting in decreased depressiveness and nervousness. However, this is a hypothesis and would only be of therapeutic value if the patient were open to exploring this idea.

When looking at all of the patients and their diverse courses of illness, emotions, and time series, it becomes clear that IBS is not expressed as a single type of IBS. Still, it is possible to draw a few conclusions for the entire group of examined patients: High same-day correlations between somatic and psychological time series (including negative coping) suggest a strong association between these complaints for all patients. This close relationship is also indicated by the day-lagged data, indicating a mutual relationship between somatic and psychological complaints. Possible physiological mechanisms linking somatic and psychological complaints in IBS patients can be explained *via* the (microbiota-) gut-brain axis. Psychological stress can lead to an activation of the autonomic system, the HPA-Axis, or the immune system resulting in increased IBS symptoms (5, 14). Vice versa, IBS-symptoms can trigger increased rumination, catastrophizing, and illness worries which is then reflected by higher same-day or lagged temporal relationships between somatic and psychological symptoms (e.g., rumination over the course of the night would lead to increased psychological stress and *via* mast cell activation to increased IBS-symptoms on the following day).

Our study has several limitations. Firstly, we examined only eight patients who suffered from IBS. As inter-individual variability can be accentuated and obliterated by a small sample size, the generalizability of the results is limited. Secondly, we analyzed associations with abdominal pain because it is the cardinal symptom of IBS. However, symptoms such as diarrhea, urgency, or the feeling of not being able to empty the bowels can be equally disturbing for the patients. The results – particularly the determined associations between coping strategies and symptoms – are therefore limited and only valid in regard to the cardinal symptom, abdominal pain. Thirdly, we were able to detect day-to-day changes only; shorter periods of time could not be captured. Nevertheless, previous studies focused mainly on longer time periods or had no longitudinal data at all. As a result, this approach is still more advantageous in terms of capturing the direct relationships. Finally, it is possible that irritable bowel symptoms and psychological variables were driven by factors that were not captured in the data set such as stressful life events that had occurred prior to the study. However, the most significant strength of our study is that we, by examining all patients individually, could show a clear picture of the patients and their differences, even if our conclusions are highly speculative. The high data density per patient is helpful as IBS is a complex illness with, in all likelihood, heterogeneous genesis and factors.

This study has several implications: Clinicians and researchers should be aware of the great variability of patients with IBS; patients may present their fears, triggers, emotions, and coping strategies in a multiplicity of ways. While some strategies may be helpful for certain patients (e.g., positive coping), this approach has no or even a reverse effect in others. Therefore, a very accurate anamnesis should always be taken, and the therapy should be adapted to the individual patient's needs. As a result, the direction of the effect is highly relevant in deciding whether therapy should focus primarily on IBS symptoms or on the psychological distress. For future research on patients with IBS it is important to be aware of the dilution and loss of data in aggregated data analysis. The varying patterns of psycho-somatic symptoms and dynamics indicate that individual factors are moderating the interdependency of psychological and somatic symptoms. Possible person-level moderators, in our sample, could be a high pressure to perform (P1, P4), illness worries (P2), neglect of feelings (P3), eating disorder (P5), social anxiety (P6), biographical stressors (P7), or resignation (P8). It could be important to more closely investigate these person-level moderators in order to generate a better understanding of the diverse mechanisms in IBS and to apply appropriate therapeutic strategies.

## Conclusion

In the IBS cases here presented, we found a high correlation between somatic and psychological complaints, as was also represented in day-lagged relationships. The patients exhibited a wide variety of their particular course of illness. Our study supports the finding that patients with IBS are a heterogeneous group, and that individualized biopsychosocial explanatory models and treatment concepts are needed.

## Data Availability Statement

The raw data supporting the conclusions of this article will be made available by the authors upon request, without undue reservation.

## Ethics Statement

The study was reviewed and approved by the Ethics Committee of the University Hospital, Heidelberg.

## Author Contributions

BW, FE, ES-K, and RS conceived and designed the study. FE, SB, ES-K, and RS collected the data. TS statistically analyzed and all authors interpreted the data. FE, BW, and TS drafted the manuscript. All authors critically revised the manuscript and provided important intellectual content and approved the final version.

## Funding

The study was supported by the Medical Faculty of the University of Heidelberg, Postdoctoral Fellowship Program. For the publication fee we acknowledge financial support by Deutsche Forschungsgemeinschaft within the funding programme Open Access Publikationskosten as well as by Heidelberg University.

## Conflict of Interest

The authors declare that the research was conducted in the absence of any commercial or financial relationships that could be construed as a potential conflict of interest.

## Publisher's Note

All claims expressed in this article are solely those of the authors and do not necessarily represent those of their affiliated organizations, or those of the publisher, the editors and the reviewers. Any product that may be evaluated in this article, or claim that may be made by its manufacturer, is not guaranteed or endorsed by the publisher.

## Abbreviations

IBS: irritable bowel syndrome
AP: abdominal pain
DI: IBS- associated daily impairment
N: nervousness
T: tension
D: depressiveness
PD: pain-associated discomfort
C: catastrophizing
H: hopelessness
CPT: coping with positive thoughts
CIP: coping with imagining pain outside the body
P: patient.
